# Levels of heavy metals in liver and kidney of dogs from urban environment

**Published:** 2012-04-21

**Authors:** F.P. Serpe, R. Russo, A. De Simone, S. Florio, M. Esposito, L. Severino

**Affiliations:** 1 *University of Naples Federico II, School of Veterinary Medicine, Naples, Italy*; 2 *Istituto Zooprofilattico Sperimentale del Mezzogiorno, Department of Chimistry, Portici (NA), Italy*

**Keywords:** Cadmium, Dog, Environment, Lead, Mercury

## Abstract

Lead, cadmium and mercury were detected in liver and kidney tissue of dogs from an urban habitat. Samples were digested in a microwave system and analyzed by atomic absorption spectroscopy. Results of the current study showed that at least one of the three heavy metals was detected in tissues of all examined dogs. These findings make us suppose that humans are exposed to the same heavy metals similar to those of dogs that are exposed since they share the same environment. Mercury concentrations detected in kidney of household dogs were higher than stray dogs, therefore the involvement of pet food in exposure to mercury can be supposed.

## Introduction

Environmental pollution by heavy metals is ubiquitous. This is due to both the natural abundance of metals within earth’s crust and human activities (Langner *et al.*, 2011; Markert *et al.*, 2011). Some of them are of great toxicological concern and have a wide range of toxic effects in both humans and animals. Toxic effects usually associated with chronic exposure are mutagenicity, carcinogenicity, teratogenicity, immuno-suppression, poor body condition and impaired reproduction (Beyersmann and Hartwig, 2008; Garcia-Leston *et al.*, 2010; Lehmann *et al.*, 2011).

Domestic and wild animals are used to assess the quality of the environment and are sentinels o f great importance for toxicological risk assessment in general. In particular, pets such as dogs and cats, who for years shared the same habitat with humans, are inevitably exposed to the same environmental pollutants (Backer *et al.*, 2001; Schmidt, 2009; Bischoff *et al.*, 2010; Reif, 2011).

Although several studies have been carried out to assess exposure of wild animals to heavy metals, there is little information on exposure to pollutants of domestic animals (Sakai *et al.*, 1995; Balagangatharathilagar *et al.*, 2006).

The aim of the current study was to detect different heavy metals in household and stray dogs living in the urban area of Naples (Italy). Lead, cadmium and mercury concentrations were measured in canine liver and kidney by atomic absorption spectroscopy.

## Materials and Methods

Necropsy of 38 dogs selected for the current study, died from different causes, was performed at the Department of Pathology and Animal Health of School of Veterinary Medicine of Naples.

The dogs were divided into household dogs (n = 19) and stray dogs (n = 19) and were subsequently divided into 4 groups based on age (group I: 1-4 years, group II: 5-8 years, group III: 9-12 years and group IV: 13-17 years). Samples of liver and kidney were collected from each animal, identified and stored at -20°C until chemical analysis.

Tissues were thawed, homogenized and then aliquots of each sample (0.50 ± 0.01 g) were digested in 4.0 ml of 70% nitric acid (Carlo Erba), 1.5 ml of 30% hydrogen peroxide (Baker Analyzed) and 3.5 ml of ultrapure water for atomic absorption spectroscopy (Best Chemicals) in a microwave digestion system (Milestone, FKW) under high pressure and temperature at 190°C.

Digested samples were analyzed for quantitative determination of heavy metals. Lead and cadmium were determined by atomic absorption spectrophotometer equipped with graphite furnace atomizer with Zeeman effect (Analyst 800, Perkin-Elmer). Mercury was determined by hydride-generation atomic absorption spectrophotometer (CV-AAS, 3110, Perkin-Elmer).

Matrix modifiers, monobasic ammonium phosphate and magnesium nitrate (1% Mg) were purchased from Perkin Elmer (US Massachusetts). Standard solutions of lead, cadmium and mercury, prepared by dilution of multi-elemental standard solutions of 1000 mg L^−1^, were purchased from Merck (Darmstadt, Germany) and working standard solutions of all trace elements were prepared by diluting stock solutions with ultrapure water. Quantification was performed by external standardization, with correction for recovery percentage. Calibration curves were obtained analysing standard solutions of each trace element. All concentration was expressed as mg kg^-1^ of wet weight.

Results represent mean value of at least three independent analyses and were processed statistically using the analysis of variance (ANOVA).

## Results

The results are summarized in Table 1. Lead was present in all examined samples at levels between 0.074 and 0.949 mg kg^-1^ (mean value = 0.321) on wet weight (w.w.) in liver and between 0.049 and 1.001 mg kg^-1^ w.w. in kidney (mean value = 0.293). Cadmium was found in all kidney samples and in 95% of liver samples, concentrations were within a range from not detectable (ND) to 0.352 mg kg^-1^ w.w. (mean value = 0.093) in liver, and between 0.011 and 0.984 mg kg^-1^ w.w. (mean value = 0.259) in kidney. Mercury was present in 71% of liver samples and in 55% of kidney samples and concentrations were found between ND and 0.418 mg kg^-1^ w.w. (mean value = 0.054) in liver, while levels were between ND and 0.328 mg kg^-1^ w.w. (mean value = 0.040) in kidney.

**Table 1:** Levels of lead, cadmium and mercury in liver and kidney (mg kg^-1^ wet weight) of all dogs from urban area (n = 38).

	Lead (Pb)		Cadmium (Cd)		Mercury (Hg)	
	
	Liver	Kidney	Liver	Kidney	Liver	Kidney
Mean value	0.321	0.293	0.093	0.259	0.054	0.040
SD	0.198	0.231	0.079	0.238	0.044	0.021
Min	0.074	0.049	ND	0.011	ND	ND
Max	0.949	1.001	0.352	0.984	0.418	0.328

**Fig. 1:**
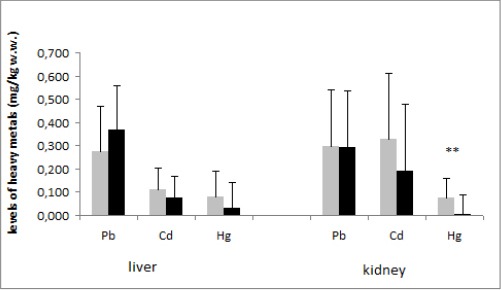
Mean values of lead, cadmium and mercury in liver and kidney of household and stray dogs (mg kg^1^ wet weight) from urban area (n = 38). **: P < 0.01 Grey: household dogs; black: stray dogs

Highest concentrations of mercury were detected in the kidney of household dogs, as reported in Fig 1.

Sex did not affect metal concentrations, while we found higher accumulation of lead and cadmium in the older dogs (13-17 years) although the difference was not statistically significant (data not shown).

## Discussion

In the present research, we examined the levels of heavy metals in dogs lived in urban areas with high anthropogenic impact and we found lead in 100% of examined tissues, cadmium in 100% of kidney and 95% of liver samples, while mercury was detected in 55% of kidney and 71% of liver samples, although we detected low concentrations of each metal.

The liver and kidney concentrations of all metals found in the present study are not considered to give rise to clinical manifestations of toxicity (Beretta, 1984; Ghisleni *et al.*, 2004; Hansmann *et al.*, 2009; Bischoff *et al.*, 2010), even if they are indicative of a chronic heavy metal exposure.

In general, we found higher concentrations of lead than the other metals, maybe for the presence of such element in the environment. Nevertheless, the environmental contamination by lead is increasingly reduced likely due to the restrictions introduced by the international legislation relating the phasing-out of leaded petrol in EU (Commission Directive 98/70/CE).

Although we found higher concentration of lead than for cadmium and mercury, the lead levels were unable to exert clinical symptoms of toxicity in canine species (Beretta, 1984; Hoff *et al.*, 1998).

López-Alonso *et al*. (2007) carried out a study about toxic metal exposure in dogs living in both urban and rural habitats and referred that lead concentrations varied from not detectable to 856 μg kg^-1^ w.w. These levels, although lower than those detected in our study, were significantly higher in the liver (57.7 μg kg^-1^ w.w.) than in the kidney (23.1 μg kg^-1^ w.w.) of examined dogs. Another study carried out in dogs from an urban environment showed that mean value of the blood lead levels were 0.113 ppm (Ghisleni *et al.*, 2004).

Cadmium concentrations were significantly highest in kidney tissue for which the metal has a particular tropism. This is supported by López-Alonso and colleagues (2007) who detected a mean cadmium concentration of 175.5 μg kg^-1^ w.w. in the kidney and 58.0 μg kg^-1^ w.w. in the liver of dogs. Cadmium concentrations in the kidney of dogs analyzed by López-Alonso *et al*. (2007) varied significantly with both age and sex, whereas we did not find such significant differences.

Regarding mercury, our results are in agreement with previously published literature (Dunlap *et al.*, 2007; López-Alonso *et al.*, 2007). In particular, our study indicates low mercury levels with highest concentrations in kidneys of household dogs. Considering that diet is the most important difference between household and stray dogs, we suppose the reasons to be, for example, fish-based wet pet foods as suggested by Sakai *et al*. (1995). On the other hand, a correlation with diet could be supposed for lead exposure as well. In fact, dogs living in urban or rural habitat fed with commercial feed showed higher liver lead residues than dogs fed with homemade feed or a mixture of commercial and homemade feed likely due to not adequately controlled canned feed (López-Alonso *et al.*, 2007). In fact, it is well known that diet could be a source of different contaminants, not only heavy metals but also polychlorinated biphenyls, pesticides and brominated flame retardants (Sonne *et al.*, 2006; 2008; 2010)

Metal exposure of animal can be derived from environmental contamination as well as from the diet (Goyer, 2000; Duran *et al.*, 2010; Kerin and Lin, 2010; Markert *et al.*, 2011).

Considering the results of the current study, we could suppose that human, living in the same area where the dogs were selected for the study, is exposed to metal contamination as well. Heavy metals concentrations detected in tissues of dogs included in the current study were generally low and unable to exert toxic effects. Nevertheless, a prolonged exposure leading to a bioaccumulation of metals in animal tissues could be expected, so chronic toxic effects could not be excluded. Such consideration can be extended to human beings who share the same environment with dogs.

There is a lack of information in the international literature regarding the usefulness of pets as bioindicators of human metals exposure and, to our knowledge, no study has been carried out to correlate the levels of heavy metals in dog tissues and human exposure. Nevertheless, according to other Authors (Backer *et al.*, 2001; Ghisleni *et al.*, 2004; Schmidt, 2009), we could consider the usefulness of pets as a sentinel model to assess human metals exposure.

In conclusion, despite the different variables, the results of the current work provide guidance to continue the study undertaken considering a wider number of animals in to both environments where animals live and to diet.

Finally, results of the higher mercury concentrations in the tissues of household dogs may be considered as a starting point to investigate the role of diet related to the exposure of pets to residual contaminants that may come from inadequately controlled raw materials. Monitoring studies in feed could be useful in order to verify whether the amount of contaminants in feed pose a risk for both human and animal welfare.
